# Loss and Gain in the Evolution of the *Salmonella enterica* Serovar Gallinarum Biovar Pullorum Genome

**DOI:** 10.1128/mSphere.00627-18

**Published:** 2019-04-03

**Authors:** Yachen Hu, Zhenyu Wang, Bin Qiang, Yaohui Xu, Xiang Chen, Qiuchun Li, Xinan Jiao

**Affiliations:** a Key Laboratory of Prevention and Control of Biological Hazard Factors (Animal Origin) for Agri-food Safety and Quality, Ministry of Agriculture of China, Yangzhou University, Yangzhou, China; b Jiangsu Key Lab of Zoonosis/Jiangsu Co-Innovation Center for Prevention and Control of Important Animal Infectious Diseases and Zoonoses, Yangzhou University, Yangzhou, China; c Joint International Research Laboratory of Agriculture and Agri-Product Safety, Yangzhou University, Yangzhou, China; d College of Veterinary Medicine, Henan University of Animal Husbandry and Economy, Henan, China; University of Wisconsin—Madison

**Keywords:** *Salmonella enterica* serovar Gallinarum biovar Pullorum, evolution, genome degradation, genomic epidemiology, multidrug resistance

## Abstract

Salmonella enterica subspecies *enterica* serovar Gallinarum biovar Pullorum (*S*. Pullorum) is the etiological agent of pullorum disease, causing white diarrhea with high mortality in chickens. There are many unsolved issues surrounding the epidemiology of *S*. Pullorum, including its origin and transmission history as well as the discordance between its phenotypic heterogeneity and genetic monomorphism. In this paper, we report the results of whole-genome sequencing of a panel of 97 *S*. Pullorum strains isolated between 1962 and 2014 from four countries across three continents. We utilized 6,795 core genome single nucleotide polymorphisms (SNPs) to reconstruct a phylogenetic tree within a spatiotemporal Bayesian framework, estimating that the most recent common ancestor of *S*. Pullorum emerged in ∼914 CE (95% confidence interval [95%CI], 565 to 1273 CE). The extant *S*. Pullorum strains can be divided into four distinct lineages, each of which is significantly associated with geographical distribution. The intercontinental transmissions of lineages III and IV can be traced to the mid-19th century and are probably related to the “Hen Fever” prevalent at that time. Further genomic analysis indicated that the loss or pseudogenization of functional genes involved in metabolism and virulence in *S*. Pullorum has been ongoing since before and after divergence from the ancestor. In contrast, multiple prophages and plasmids have been acquired by *S*. Pullorum, and these have endowed it with new characteristics, especially the multidrug resistance conferred by two large plasmids in lineage I. The results of this study provide insight into the evolution of *S*. Pullorum and prove the efficiency of whole-genome sequencing in epidemiological surveillance of pullorum disease.

**IMPORTANCE** Pullorum disease, an acute poultry septicemia caused by *Salmonella* Gallinarum biovar Pullorum, is fatal for young chickens and is a heavy burden on poultry industry. The pathogen is rare in most developed countries but still extremely difficult to eliminate in China. Efficient epidemiological surveillance necessitates clarifying the origin of the isolates from different regions and their phylogenic relationships. Genomic epidemiological analysis of 97 *S*. Pullorum strains was carried out to reconstruct the phylogeny and transmission history of *S*. Pullorum. Further analysis demonstrated that functional gene loss and acquisition occurred simultaneously throughout the evolution of *S*. Pullorum, both of which reflected adaptation to the changing environment. The result of our study will be helpful in surveillance and prevention of pullorum disease.

## Abstract (precis)

Pullorum disease, an acute poultry septicemia caused by *Salmonella* Gallinarum biovar Pullorum, is fatal for young chickens and is a heavy burden on poultry industry. The pathogen is rare in most developed countries but still extremely difficult to eliminate in China. Efficient epidemiological surveillance necessitates clarifying the origin of the isolates from different regions and their phylogenic relationships. Genomic epidemiological analysis of 97 *S*. Pullorum strains was carried out to reconstruct the phylogeny and transmission history of *S*. Pullorum. Further analysis demonstrated that functional gene loss and acquisition occurred simultaneously throughout the evolution of *S*. Pullorum, both of which reflected adaptation to the changing environment. The result of our study will be helpful in surveillance and prevention of pullorum disease.

## INTRODUCTION

Salmonella enterica subspecies *enterica* serovar Gallinarum biovar Pullorum (*S*. Pullorum) can produce pullorum disease, an acute systemic disease specific to poultry which mainly infects young birds and causes high mortality. Pullorum disease once wrought havoc with the poultry industry worldwide and led to substantial economic losses in the early 20th century. Owing to the introduction of pullorum-typhoid programs based on detection and elimination of affected birds, pullorum disease has been eradicated from commercial poultry in most developed countries, but outbreaks still occur frequently in other regions (1, 2). In China, frequent outbreaks of pullorum disease affect nearly every province and are accompanied by high infection rates and increasing antimicrobial resistance (AMR). *S*. Pullorum strains isolated after 2000 in China were found to exhibit a high level of resistance to multiple antibiotics, including ampicillin, nalidixic acid, streptomycin, tetracycline, trimethoprim, and sulfafurazole (3). Although multidrug resistance was presumed to be related to a class 1 integron (4), the details of the molecular mechanisms remain unclear.

Recently, the phylogenetic relationship between *S*. Pullorum and its relatives has been investigated using genomic analysis (5). *S*. Pullorum and *S*. Gallinarum biovar Gallinarum (referred to here as *S*. Gallinarum) share an ancestor which was a direct descendant of *S*. Enteritidis. In epidemiological investigations, it is necessary not only to distinguish *S*. Pullorum from its relatives but also to subtype it at an isolate-specific level. As with other highly clonal organisms, *S*. Pullorum isolates exhibit limited genetic diversity, which frustrated traditional typing methods, such as multilocus enzyme electrophoresis (MLEE), ribotyping, and pulsed-field gel electrophoresis (PFGE) (6–8), due to low resolution. Repeat-based approaches, such as clustered regularly interspaced short palindromic repeat (CRISPR) typing, slightly improved resolution (9), but they were assumed to be less phylogenetically correlative due to horizontal gene transfer (HGT) (10).

In contrast to their genetic homogeneity, *S*. Pullorum strains exhibit phenotypic heterogeneity. Notably, *S*. Pullorum strains could be subdivided into aerogenic and anaerogenic groups using multiple biochemical markers (11). The former were able to ferment rhamnose and xylose, while the latter were not. Another example is the variation of O-antigen 12 subfactors; the standard strains of *S*. Pullorum mainly express 12_3_, while the variant strains mostly express 12_2_ (1). The nature of the genomic mutations affecting phenotype and the reliability of these mutations for clustering epidemiologically important strains remain to be investigated.

With the development of next-generation DNA sequencing (NGS) technology, the cost of sequencing has plummeted (12). At present, it is possible to perform whole-genome sequencing (WGS) on large-scale bacterial isolates, a technology which has created an era of “genomic epidemiology” (13, 14). By analyzing WGS data, two main goals can be archived simultaneously: (i) inferring the phylogenic relation between bacterial isolates at single-base-level resolution and (ii) illuminating the genetic mechanism of epidemiologically important phenotypes (13). In order to reconstruct the population structure and evolutionary history of *S*. Pullorum, we performed WGS on a collection of 97 *S*. Pullorum strains that had been isolated in China, Brazil, the United Kingdom, and Denmark between 1962 and 2014. Core-genome single-nucleotide polymorphisms (SNPs) were called and used to infer the phylogeny in a spatiotemporal Bayesian framework within which the loss of functional genes and the acquisition of mobile genetic elements (MGEs) were further analyzed. In this context, we explored the evolutionary and transmission history of *S*. Pullorum, as well as the genetic mechanisms of biochemical traits and drug resistance in this organism.

## RESULTS

### Phylogeny, evolutionary dynamics, and transmission history.

After removing genomic regions identified as repeats, deletions, or recombinations, we obtained a 4,160,759-bp core genome from the reference genome of *S*. Pullorum RKS5078. The regions of different forms overlapped with each other. The recombination events recognized by Gubbins (15) are mainly false positives caused by interference of repeats and deletions, indicating that homologous recombination rarely or never occurred in *S*. Pullorum since the emergence of its most recent common ancestor (MRCA). A total of 6,795 SNPs in the core genome (termed core genome SNPs []cgSNPs]) were identified, representing slightly more than the number seen in other genetically monomorphic *Salmonella* serotypes, such as the 4,887 SNPs of *S*. Enteritidis (16) and the 4,525 SNPs of *S*. Paratyphi A (17).

**FIG 1:**
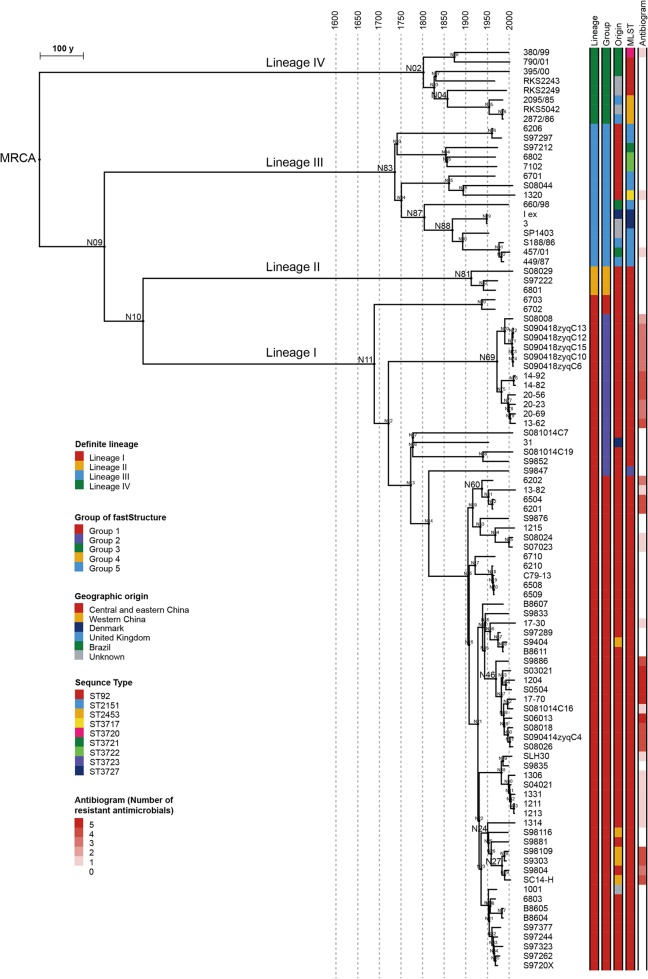
Phylogeny based on a spatiotemporal Bayesian framework. The figure shows a maximum clade credibility tree (relaxed lognormal clock; constant population size) based on 6,795 core genome SNPs, indicating four definite lineages. The length of branches represents evolutionary time, and the dashed line perpendicular to the branches indicates the age intervals in 50-year increments. The names of important nodes are enlarged. The leftmost color strip indicates the lineage as follows: I, red; II, orange, III, blue; IV, green. Other color strips show information about the fastSTRUCTURE groups, geographic origins, multilocus sequence types (MLST), and antibiograms (predicted by genotypes).

The maximum likelihood (ML) and maximum clade credibility phylogenetic trees, constructed on the basis of the cgSNPs, exhibit the same topology (see Fig. S1 in the supplemental material). Four definite monophyletic branches, designated here lineages I to IV, were revealed from both trees (Fig. 1). Reference strain RKS5078 belongs to lineage IV and is basal to all other strains in this lineage (Fig. S1). The lineages correspond to the population groups identified by fastSTRUCTURE (18) (Fig. 1; see also Fig. S1), except that the latter subdivides lineage I into two groups, which are not monophyletic as revealed by the phylogeny. The sequencing strains exhibit nine multilocus sequence types (STs), six of which were identified for the first time (Fig. 1). The new STs were submitted to Enterobase (http://enterobase.warwick.ac.uk/) and assigned identifiers of ST3717, ST3720-ST3723, and ST3727. According to the phylogeny, the diverse STs do not provide effective discrimination, since the strains of lineage I, II, and IV all belong to ST92 and its single-locus variants (SLVs). The only apparent correspondence is that the strains of lineage III all belong to either ST2151 or its SLVs. It is evident that the typing method based on analysis of seven housekeeping genes (19) exaggerates the genetic distance between the strains of different STs, with the exaggeration possibly caused by the presence of random mutations in the limited number of genes selected.

10.1128/mSphere.00627-18.1 FIG S1 Maximum likelihood (ML) tree based on 6,795 cgSNPs. Reference strain RKS5078 of *S*. Pullorum was added to the ML tree and is indicated in blue. Download FIG S1, TIF file, 0.8 MB. Copyright © 2019 Hu et al. 2019 Hu et al. This content is distributed under the terms of the Creative Commons Attribution 4.0 International license.

**FIG 2:**
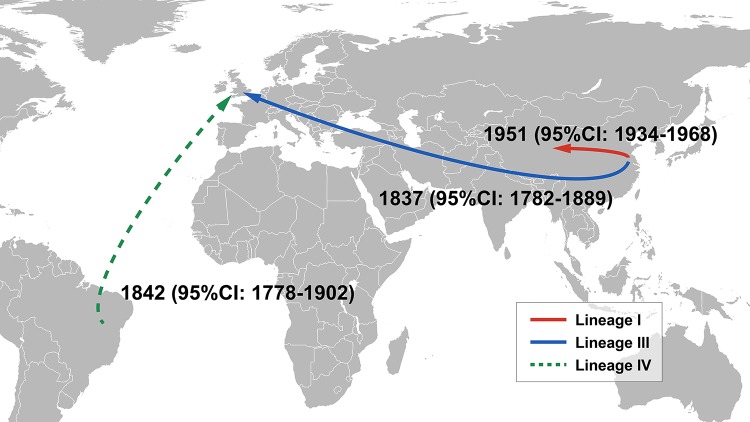
Geographic transmissions. Arrows represent the transmission paths of three lineages with mean dates and 95% confidence intervals.

Both marginal-likelihood estimation methods (the path sampling method and the stepping-stone sampling method) determined that the best Bayesian model in this case consists of the combination of constant population size with a relaxed lognormal clock, and that approach resulted in an estimation that the most recent common ancestor of *S*. Pullorum appeared in 914 CE (95% confidence interval [95%CI], 565 to 1273 CE), approximately 1,100 years ago. The mean mutation rate of the entire population was estimated to be 1.92 × 10^−7^ substitutions/site/year or 0.80 SNPs/genome/year, comparable with the rates determined previously for *S*. Enteritidis (2.2 × 10^−7^), *S*. Paratyphi A (1.94 × 10^−7^), and *S*. Typhimurium DT104 (2.79 × 10^−7^) (16, 17, 20). The estimated emerging dates of the descendant nodes and mutation rates of the clades are listed in Table S3 in the supplemental material. All four lineages diverged after the late 17th century (Fig. 1; see also Table S3). It is obvious that there is an association between the lineages and the geographical distribution of the strains. Lineages I and II are unique to China, with only one exception, namely, strain 31, which was isolated in Denmark. The original date of isolation of strain 31 is unproven but, using the temporal Bayesian framework, was estimated to have been 1952. One clade of lineage I spread to Western China (N24 to N25) in 1951 (95%CI, 1934 to 1968) (Fig. 2). Lineage III is presumed to have originated in China on the basis of the geographic distribution of the deeper clades. One of the branches disseminated overseas and subsequently entered Europe (N87 → N88). The transmission event can be dated to 1837 (95%CI, 1782 to 1889) (Fig. 2) and was likely associated with the “Hen Fever” that swept Europe and the United States in the mid-19th century (21). Lineage IV is presumed to have originated in the Americas. Coincidentally, lineage IV was also introduced to Europe (N03 → N04) in the mid-19th century (1842; 95%CI, 1778 to 1902) (Fig. 2). However, due to the lack of geographical information about the related strains, it is not possible to speculate about its exact transmission route.

**FIG 3:**
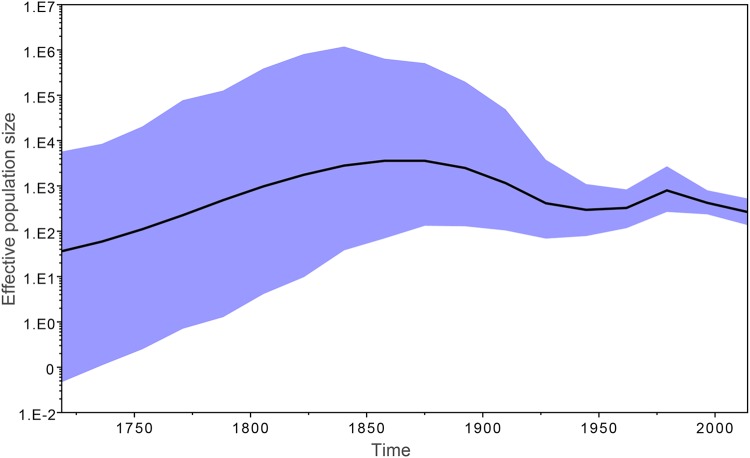
Bayesian SkyGrid plot showing the changes in effective population size (*N*_e_) of lineage I through time. The black curve represents mean values and the cyan 95% confidence intervals.

For the entire population, the best model assumes a constant effective population size (*N*_e_), which is not applicable to all local clades. By performing an independent model comparison analysis of lineage I, the predominant lineage in China, it was found that the Bayesian SkyGrid model outperformed the others. As shown in Fig. 3, in about 150 years after the emergence of MRCA, the *N*_e_ of lineage I increased about 100-fold. The *N*_e_ declined dramatically between the 1850s and the 1950s, during which period China suffered a “century of humiliation,” and the poultry industry was likely to have been affected seriously by the wars. The reason for the decline of the *N*_e_ is likely to have been the absence of hosts in which it could survive, considering the host specificity of *S*. Pullorum. Subsequently, the *N*_e_ rebounded briefly and dropped again in the 1970s, possibly due to the introduction of the use of antibiotics in poultry farming. The supporting evidence suggests that all of the multidrug-resistant clades are distributed in lineage I (Fig. 1) and that most of them arose after the 1970s (Table S3), indicating the existence of an antibiotic-selective environment.

### Functional gene loss attributable to pseudogene formation and gene deletion.

**FIG 4:**
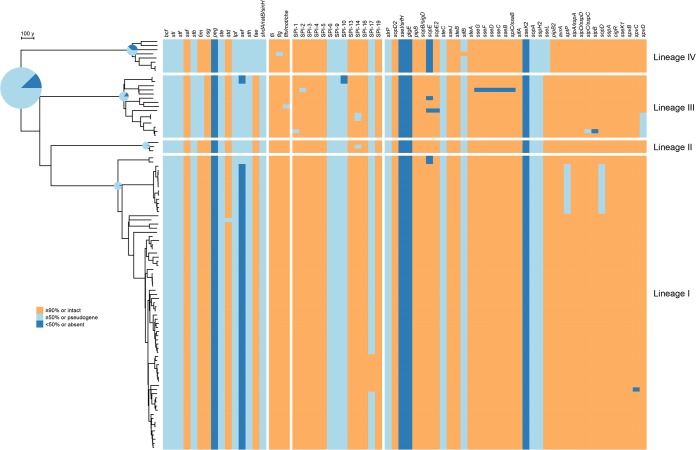
Heat map indicating the integrity of virulence factors coupled with phylogeny. The numbers of functionally inactivated genes accumulated at the nodes of root and the ancestors of four lineages are indicated by the relative sizes of pie charts corresponding to the phylogeny (left). Within the pie charts, the proportions of two forms of gene loss (pseudogenes and gene deletions) are represented by light blue and dark blue, respectively. The integrity of 14 fimbrial operons, alongside one cluster of nonfimbrial adhesin genes, three flagellar clusters, 13 *Salmonella* pathogenicity islands, and 38 effectors of type III secretion systems, is depicted by color blocks in the heat map (right) as follows: orange for intact genes or gene clusters with ≥90% intact gene ratios (IGR), light blue for pseudogenes or ≥50% IGR clusters, and dark blue for absent genes or <50% IGR clusters.

After mapping the profiles of pseudogenes and deleted genes in each isolate in the phylogeny, we defined the extent of the genome degradation of the MRCA nodes in the tree (Fig. 4). About 80% of the degraded genes are common to all *S*. Pullorum strains (not including the ancestral pseudogenes present in *S*. Enteritidis and the absent genes of prophages φSE10 and φSE20 which are specific to *S*. Enteritidis PT4 [22]) and include 78 shared by *S*. Pullorum and *S*. Gallinarum (with identical mutation sites) and 151 accumulated dependently by the MRCA of *S*. Pullorum (see Table S4 for a full list). Table S5 summarizes the roles played by these genes. Inactivation of these genes affects 23 metabolic pathways and transport systems, some of which have previously been described, such as arginine degradation (*astA*), putrescine biosynthesis (*speC*), glycogen biosynthesis (*glgB*) and degradation (*glgX*), and tetrathionate respiration (*asrAB*, *ttrB*) (22, 23). Trimethylamine N-oxide (TMAO) can be used as a terminal electron receptor for anaerobic respiration in *Salmonella* and as support for the anaerobic growth of *Salmonella* on glycerol (24). *S*. Pullorum harbors a deletion mutation in the *tor* operon, which affects the product of reductase (*torA*), *c*-type cytochrome (*torC*) and their two-component regulation system (*torR*/*torT*). TMAO, an osmolyte, is abundant in the tissues of marine animals, protecting against the protein-destabilizing effects of pressure (25). The inactivation of the *tor* operon in *S*. Pullorum suggests that it is dispensable for survival in the chicken host. In addition, *S*. Pullorum inactivates genes encoding components of two of three osmoprotectant transport systems: ProU (*proW* encoding transporter permease) and OsmU (*osmV* encoding transporter ATP-binding protein) (26). The only intact transporter, ProP, may become necessary for uptake of osmoprotectants, such as betaine. This also confirms the standpoint assumed by Langridge (5), according to which reduction in the redundancy of functional pathways is more important than loss of function in host adaptation. However, this may be one of the reasons why *S*. Pullorum is less resistant in the environment outside the host.

The genome degradation of *S*. Pullorum is ongoing. Lineages I, II, III, and IV have 42, 41, 53, and 60 genes, respectively, that have degraded since diverging from the MRCA. Some of these inactive genes are involved in the “chain reaction” of pathway degradation, i.e., the further degradation of related genes of a primarily inactive pathway. For instance, as mentioned above, the in-frame deletion in the pivotal gene of arginine N-succinyltransferase (AST) (*astA*) leads to the degradation of l-arginine via the AST pathway in the MRCA of *S*. Pullorum. In lineage I and lineage II, *astC* and *astB*, the genes involved in the subsequent steps of this pathway, are frameshifted. It is almost impossible for the related genes to remain intact without the selective constraints of the functional pathways. Inactivation of pathways also occurs in different lineages. There are three inactivated pathways related to carbohydrate degradation in lineage III, namely, glycerol degradation, l-rhamnose degradation, and d-galactose degradation, resulting from the inactivation of *glpK*/*glpX*, *rhaA*, and *yihR*. The impact of the pseudogene formation (nonsense mutation of Trp83*) of *rhaA* was identified by the biochemical assay (Fig. S2), which indicated that the isolates of lineage III were unable to ferment l-rhamnose. Notably, the inactive *rhaA* gene appears to be “repaired” by reversion of the premature termination codon to a lysine codon in the lineage III isolate 7102. Spontaneous reversion of pseudogenes in nature has rarely been reported. Another infrequent but noteworthy phenomenon is the homoplasy in pathway degradation, that is, independent inactivation in the same gene or in analogous functional genes or pathways among different lineages. For instance, lineage II and lineage III both have inactivated putrescine degradation pathways. Inactivation of the former is achieved by disrupting the putrescine aminotransferase gene (*patA*), and inactivation of the latter is achieved by truncating the γ-aminobutyraldehyde dehydrogenase gene (*patD*). Lack of putrescine degradation pathways makes bacteria grow defectively under various stress conditions, including oxidative stress, high temperature, and sublethal antibiotic concentrations (27). The homoplastic degradation is speculated to be caused by convergent evolution within the same selective environment, such as the same host niche (28).

10.1128/mSphere.00627-18.2 FIG S2 Comparative biochemical assays of Lineage III strain 6802 and Lineage I strain B8604 performed by API 20E. The fermentation tubes of L-rhamnose are indicated by red boxes. Blue is negative and yellow is positive. Download FIG S2, TIF file, 0.3 MB. Copyright © 2019 Hu et al. 2019 Hu et al. This content is distributed under the terms of the Creative Commons Attribution 4.0 International license.

### Distribution and integrity of virulence factors.

In addition to metabolism-related gene losses, virulence gene losses are common in *S*. Pullorum and the rates of such losses differ among the lineages (Fig. 4). *Salmonellae* possess multiple fimbriae, which are critical for epithelial cell adhesion and intestinal colonization (29, 30). As seen with the metabolic pathways, multiple fimbrial operons are degraded in *S*. Pullorum, and that process may be involved in the loss of ability to colonize in an intestinal environment. Among the 13 fimbrial operons inherited from *S*. Enteritidis, *saf*, *csg*, and *std* remain intact in almost all the *S*. Pullorum strains. Inactivating mutations exist in the genes within the remaining 10 fimbrial operons, 6 of which occurred before diverging from *S*. Gallinarum, including mutations of *sti*, *stf*, *stb*, *lpf*, *sef*, and *sth*. Within the *peg* operon, three (*pegB*-*pegC*-*pegD*) of the five genes are disrupted in all strains of *S*. Pullorum, which indicates that they are unnecessary in establishing chicken infection, despite evidence suggesting that *pegA* was established to be important in the colonization of avian intestines for *S*. Enteritidis (31). In lineage III, *fimA* and *fimI* were deleted, which may prevent the assembly of type 1 fimbriae. Like *S*. Gallinarum, *S*. Pullorum possesses a plasmid-borne *fae* fimbrial operon, which is mutually exclusive with the *pef* operon found in the virulence plasmid of *S*. Enteritidis (32). The *fae* operon is intact in all *S*. Pullorum strains, other than strains of lineage IV, within which the *faeJ* is frameshifted. There are three nonfimbrial adhesin genes in chromosomal genetic island CS54 which are involved in intestinal persistence and shedding (33). Among those three genes, the *ratB* gene was ancestrally disrupted (before diverging from *S*. Enteritidis) and *shdA* and *sinH* are intact in all *S*. Pullorum strains, while all of the three are inactivated in *S*. Gallinarum.

Both *S*. Pullorum and *S*. Gallinarum are nonmotile and nonflagellate. This status is thought to have arisen before their divergence and to have resulted from the presence of the pseudogenes of *flhB* and *flgK* (6, 34), as supported by the present study. Regardless of the random occurrence of mutations in one or two strains, the only inactivated flagellar gene is *fliP*, which is frameshifted in lineage II. It is notable that the vast majority (47 of 50) of flagellar genes remain intact in almost all strains, which suggests that they are under selection by functions other than motility.

*Salmonella* pathogenicity islands (SPIs) contribute to virulence and host interactions. In *S*. Pullorum, the SPIs, specifically, SPI-1 to SPI-5, are mostly conserved across all the lineages and appear to have been acquired in the early stage of S. enterica evolution (35), and SPI-19 encodes a type VI secretion system (T6SS) and contributes to *S*. Gallinarum survival within macrophages and aids with its colonization in chickens (36, 37). Large-scale genomic deletion occurred only once in SPI-1 and SPI-2 of strains 457/01 and 6802, respectively. Degeneration of SPI-10 occurred in both *S*. Pullorum and *S*. Gallinarum, mainly due to the pseudogene formation within the *sef* operon. Another two small SPIs, SPI-9 and SPI-17, have also been affected by pseudogenization across the four lineages. SPI-9 has lost the coding DNA sequence (CDS) *SEN_RS13585*, which encodes a type I secretion system permease, while SPI-17 has an inactivated *gtrC*, which is a bacteriophage-borne O-antigen acetyltransferase gene. Remarkably, the pseudogene reversion happened again in *gtrC* of N38, and in two other strains, by reinsertion of one of the eight consecutive adenines at positions 1560 to 1567.

Multiple type three secretion system (T3SS) effectors have been introduced by prophages. Both *S*. Pullorum and *S*. Gallinarum have lost several effectors due to the absence of prophages, including *sseI* and *gtgE* in φSE10 as well as *sseK2* in φSE20. Moreover, the *slrP*, *steC*, *sifB*, *sopA*, and *sspH2* genes have been found to be inactivated in all the strains of *S*. Pullorum, except for the reverted *sifB* gene in strain RKS2243. Lineage-specific degradation occurred only in lineage IV, where *sopE* is absent via the partial deletion of φSE12 and *sseL*, which are disrupted by a frameshift. Two additional effectors, *sptP* and *sopD*, are inactivated in N69, a clade of lineage I. Effectors have been proved to be associated with modulation of gut inflammation (38, 39) and host adaptation (40). Losses of effector proteins might correspond to the invasive lifestyle and host specificity of *S*. Pullorum.

### Characteristics of diverse prophages and the origin of prophage ST104.

In *Salmonella*, the presence of prophages diversifies the genome and potentially changes bacterial phenotypes through lysogenic conversion (41). Comparative genome analysis identified four full-length prophages distributed across all the lineages (not including the ancestral prophage remnants inherited from *S*. Enteritidis) (22), named φSPU1 to φSPU4, one of which belongs to genus *P22virus* and the other three to genus *P2virus*. The main characteristics of these prophages are summarized in Table 1. The distributions of prophages are loosely correlated with lineages, where φSPU1 mainly exists in lineage I and lineage III and φSPU3 is concentrated in lineage IV and lineage II. The other two prophages are isolate specific and clade specific, respectively.

**TABLE 1:** Characteristics of mobile genetic elements^*a*^

MGE	Size (kb)^*b*^	% G+C content	Insertion site	Type	Analogue (% identity^*c*^)	Distribution (% prevalence rate)	Cargo genes and functions
Prophages							
φSPU1	41.5	47.5	*thrW*	P22	ST104 (100)	Lineage I (93); lineage III (53); lineage II (33)	Virulence, *pipB3*; LPS modification, *gtrA*, *gtrB*, *gtrC*
φSPU2	31.1	52.7	*yfiN-rplS*	P2	PsP3 (90)	6702^*d*^	
φSPU3^*e*^	33.5	51.6	*ssrA*	P2	Fels-2 (80)	Lineage IV (100); lineage II (100); lineage III (27); lineage I (1)	
φSPU4	29.6	53.2	*yiiP-cpxP*	P2	P2 (97)	N69^*d*^	

Small plasmids							
pSPUS1	4.1	51.9		ColE1	ColE1 (98)	Lineage I (99); lineage II (67); lineage IV (63); lineage III (47)	Virulence, *ipaJ*
pSPUS2	2.3	47.4		ColE1^*f*^	ColE1 (83)	Lineage I (94); lineage IV (88); lineage II (67); lineage III^*g*^ (60)	
pSPUS3	2.5	53.6		RCR	pVCM01 (99)	Lineage III (100); lineage IV (88); lineage I (87)	

AMR plasmids							
pSPUR1	47.5	52.1		IncN	R46 (100)	N46^*d*^,h	Resistance, *dfrA17*, *aadA5h*, *sul1*, *qacE*, *tet(A)*; RMS, *ecoRIIR*, *ecoRIIM*
pSPUR2	73.2	47.6		IncX1	pOLA52 (98)	N69^*d*^	Resistance, *ars*, *sul2*, *bla_TEM-1b_*, *tet(A)* *h*
pSPUR3	48.4	46.7		IncX1	pOLA52 (95)	N27^*d*^	Resistance, *bla_TEM-1b_*, *tet(A)* *h*
pSPUR4	8.7	61.0		IncQ1	RSF1010 (100)	N27^*d*^	Resistance, *sul2*, *strAB*
pSPUR5	11.0	61.7		IncQ1	RSF1010 (100)	N60^*d*^^,^^*h*^	Resistance, *sul2*, *strAB*, *tet(A)*

a RCR, rolling-circle replication; AMR, antimicrobial resistance; RMS, restriction-modification system.

b Data represent prophage sizes, including flanked direct repeats (DRs) within the bacterial attachment site (attB).

c Values represent global sequence identity for prophages and replicon sequence identity for plasmid.

d The indicated strain belongs to lineage I.

e The indicated MGEs are diverse among the lineages.

f The indicated type is defective with respect to mobility, i.e., does not carry *mob* genes.

g The indicated MGE was integrated into the chromosome within two strains.

h The indicated element was lost in some strains.

**FIG 5:**
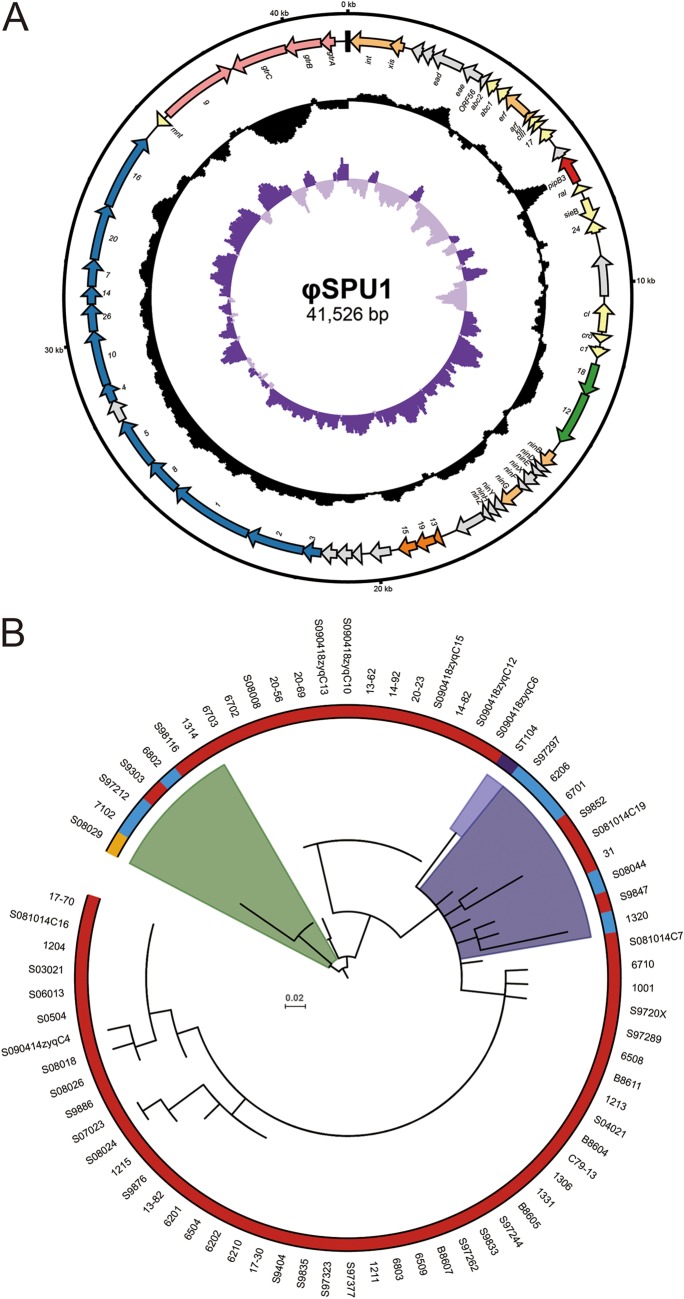
P22-like prophage φSPU1. (A) Genome map of prophage φSPU1. From the outside in, circle 1 shows sizes in kilobase pairs. Circle 2 shows the locations of genes, with the arrow indicating transcribed direction. The gene modules are color-coded according to their functions as follows: lysogeny, coral; transcriptional regulation, yellow; DNA replication, green; lysis, orange; morphogenesis and packaging, blue; LPS binding and modification, pink; unknown, gray. The putative virulence factor *pipB3* is colored red, and the black box at the top indicates the putative *att* sites. Circle 3 and circle 4 show GC content and GC skew, respectively. (B) Phylogeny of prophage φSPU1. The peripheral color strip corresponds to the lineages of the strains as follows: I, red; I, orange; III, blue; ST104 from *Salmonella* Typhimurium, purple. Colored clades in the phylogeny indicate two putative origins.

φSPU1 is 41,526 bp in length, with an average G+C content of 47.48%, which is slightly lower than that of the entire genome (52.2%) (42). The attachment site (*attB*) of φSPU1 is located within threonyl tRNA gene *thrW*, consistent with *S*. Typhimurium bacteriophage P22 (43). The genome map of φSPU1 is shown in Fig. 5A. Analysis of φSPU1 revealed 60 open reading frames (ORFs), which consist of functional genetic modules, including modules corresponding to lysogeny, transcriptional regulation, DNA replication, lysis, morphogenesis, and lipopolysaccharide (LPS) modification. Like P22, φSPU1 contains a glucosyltransferase operon, *gtrABC*. The *gtrABC* of P22 induces O-antigen conversion by conferring antigen O1 to the host (43), which suggests that the *gtrABC* of φSPU1 is also functional, because of the high identity of their products (average of 99.6%). We also identified a potential virulence gene, designated *pipB3*, the putative product of which shows sequence similarity to the T3SS effector proteins PipB and PipB2. PipB3 is 244 amino acids (aa) in length, with a predicted molecular mass of 26.7 kDa. Like PipB and PipB2, PipB3 contains tandem pentapeptide repeats, a motif which is required for efficient recruitment of kinesin-1 (44). The pentapeptide motif in PipB3 is strictly conserved, with the consensus sequence of A(N/D)(L/M/F)XX (Fig. S3), and the number of pentapeptides in different strains ranges from 7 to 27, with the highest frequency being 7 pentapeptides (*n* = 39) followed by 21 (*n* = 30).

10.1128/mSphere.00627-18.3 FIG S3 Alignment of pentapeptide repeats in three PipB-like proteins. Download FIG S3, TIF file, 0.3 MB. Copyright © 2019 Hu et al. 2019 Hu et al. This content is distributed under the terms of the Creative Commons Attribution 4.0 International license.

ST104 is a P22-like prophage shared by all the isolates of *S*. Typhimurium definitive phage type 104 (DT104) (45). φSPU1 prophages display extremely high sequence identity with ST104 (average nucleotide identities of 99.8%), so the two can be considered the same prophage. An NCBI database search obtained only 20 matched records (with coverage and identity levels of >99%) of bacterial genome assembly. *In silico* serotyping by SISTR (46) revealed that these genomes are derived from the serovars of *S*. Typhimurium and *S*. Pullorum only (with the only exception being strain 92-0392, which is likely to be *S*. Typhimurium but was mistakenly labeled *S*. Enteritidis), which made us interested in its origin. In order to explore their phylogenetic relationship, we constructed a maximum likelihood tree based on the core genome of φSPU1 and ST104 (DDBJ accession number AB102868) (Fig. 5B). From this tree, it was inferred that the phylogenic relationship of φSPU1 in lineage I is almost identical with that of its host, which suggests a vertical inheritance. The only exception is N24, a clade from Western China, whose φSPU1 prophages were horizontally acquired from an independent origin (indicated in green in Fig. 5B). Interestingly, some of the lineage III strains (N94) also inherited φSPU1 from this cryptic origin. Another origin (blue) of the φSPU1 of lineage III is located within N13, a descendant clade of lineage I, from which lineage III obtained φSPU1 twice independently (N96 and N85). It is striking that ST104 was likely acquired from this origin, too, as evidenced by the small phylogenic distance, and that idea is also supported by the fact that the MRCA of DT104 was estimated to have emerged in ∼1948 (20), much later than that of N13 (∼1772) (Table S3).

### Plasmids contribute to multidrug resistance.

**FIG 6:**
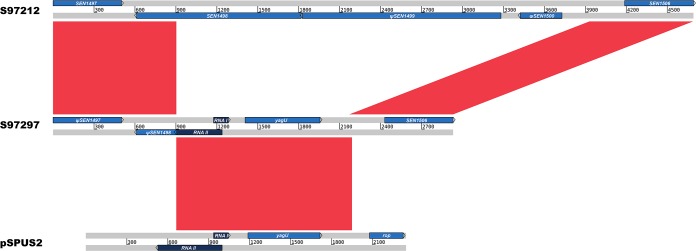
Genomic rearrangement of ROD14 introduced by the integration of pSPUS2. Compared with related strain S97212 (top), *SEN1498* to *SEN1500* in strain S97297 (middle) were replaced by a partial pSPUS2 plasmid (bottom). Red blocks indicate the homologous regions, and genes are labeled as arrows and colored blue (coding sequences) and dark blue (noncoding RNAs).

In addition to the conserved virulence plasmid present in all strains (32), multiple plasmids were identified in *S*. Pullorum, including three small plasmids of <5 kb (designated pSPUS1 to pSPUS3) and five large plasmids involved in antimicrobial resistance (AMR) (designated pSPUR1 to pSPUR5) (Table 1). The three small plasmids are relatively conserved across all four lineages, which suggests that they had been acquired by the MRCA of *S*. Pullorum and were lost in subsequent evolution. We hypothesized that the loss of small plasmids may occur in the occasional events in evolution, such as integration with the chromosome or external plasmids, followed by removal of purifying selection. There is evidence in strains 6206 and S97297, where a partial pSPUS2 plasmid was inserted between genes *SEN1497* and *SEN1506* and deleted or truncated genes *SEN1498* to *SEN1500* simultaneously (Fig. 6). Notably, this genomic rearrangement was found to be located in ROD14, a mosaic region which was degraded to various degrees in *S*. Enteritidis and *S*. Gallinarum (22).

Small plasmids were previously used for subtyping *S*. Pullorum (47). As shown in Fig. S4, there was a loose correlation between loss events and lineages in that pSPUS1 and pSPUS2 were found to be more highly conserved in lineage I. There has been little research on the functions of the small plasmids. An exception is pSPUS1, previously named pSPI12, which highly expresses putative virulence gene *ipaJ* in *S*. Pullorum infection (48).

10.1128/mSphere.00627-18.4 FIG S4 Distribution of mobile genetic elements, including four prophages, three small plasmids, and five resistance plasmids. Download FIG S4, TIF file, 0.5 MB. Copyright © 2019 Hu et al. 2019 Hu et al. This content is distributed under the terms of the Creative Commons Attribution 4.0 International license.

**FIG 7:**
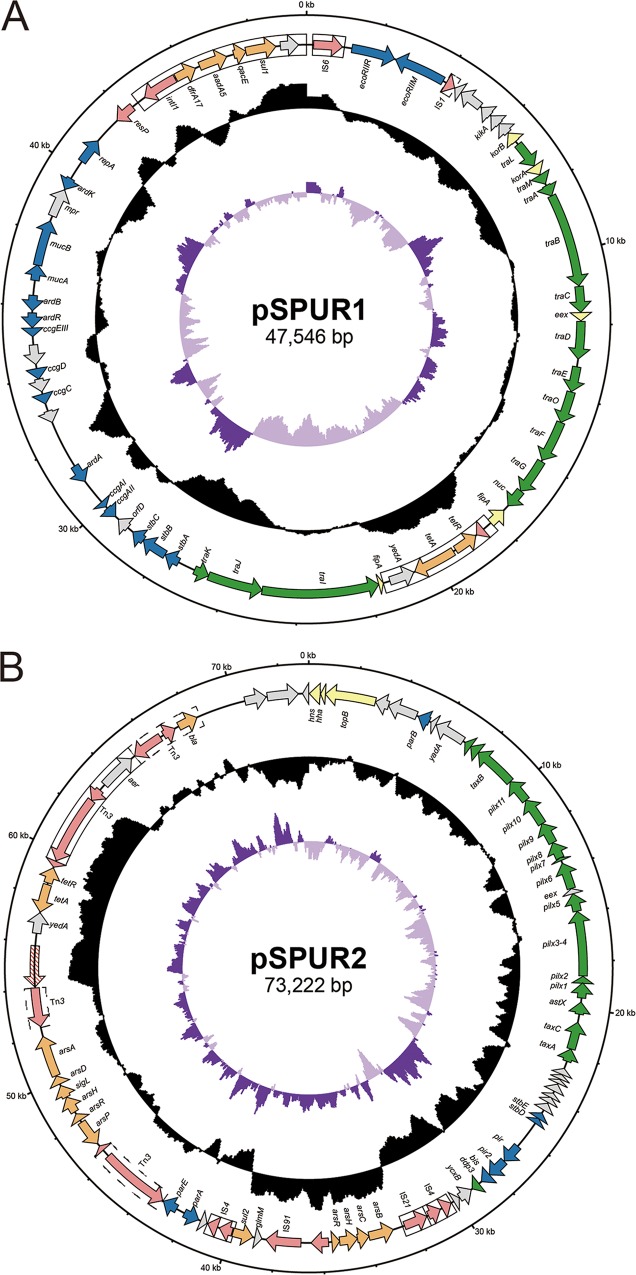
Multidrug resistance plasmids. (A) Genome map of IncN plasmid pSPUR1. The functional genes are color-coded as follows: maintenance and stability, blue; conjugal transfer, green; transcriptional regulation, yellow; transposases and resolvases, pink; resistance, orange; other functions, gray. (B) Genome map of IncX1 plasmid pPSUR2. The genes are color-coded as described for pSPUR1 in the panel A legend.

The five AMR plasmids belong to three incompatibility groups, IncN, IncX1, and IncQ1, among which pSPUR1 and pSPUR2 are most important, due to their broad prevalence and genetic complexity (Fig. 7). pSPUR1 is an IncN plasmid, 47,546 bp in length, with an average G+C content of 52.1%. PlasmidFinder (49) analysis determined that the replicon of pSPUR1 has an identity of 99.7% with that of R46, the prototypical plasmid of IncN. They have similar plasmid backbones encoding the core functions of the plasmid, including conjugal transfer (*tra*), stability (*stb*), antirestriction (*ard* and *ccg*), and mutagenesis enhancement (*muc*). pSPUR1 contains a large class 1 integron carrying the *intI1* integrase gene and five sequential resistance cassettes: dihydrofolate reductase gene *dfrA17*, conferring trimethoprim resistance; aminoglycoside-(3′′)-adenylyltransferase gene *aadA5*, conferring streptomycin and spectinomycin resistance; dihydropteroate synthase gene *sul1*, conferring sulfonamide resistance; multidrug efflux pump gene *qacE*, conferring resistance to quaternary ammonium compounds, and a putative GNAT family N-acetyltransferase gene. Notably, the presence of *aadA5* is not sufficient to induce high resistance to streptomycin in MIC assays (Table S2). The genotypic and phenotypic discrepancies with respect to the effects of the *aadA* gene also appear in Escherichia coli and is attributed to the exorbitant breakpoint (50). The *aadA5* gene cassette also has been lost in a minor terminal clade, which may be due to purifying selection. Moreover, a putative insertion sequence (IS) associated with tetracycline resistance, designated IS*spu1*, inserts within the *fipA* gene and disrupts it. This cryptic IS carries four genes, including a putative relaxase gene; tetracycline efflux pump gene *tetA* and corresponding repressor gene *tetR*, which are possibly derived from Tn*1721* (>99.7% protein identity); and putative inner membrane transporter gene *yedA*. There is a pair of almost perfect (only one base mismatched) 247-bp inverted repeats (IRs) on the two ends of IS*spu1*, and those IRs are flanked by a pair of 8-bp direct repeats (DRs) (5′-CCAGCAAG-3′). However, the transposition mechanism of IS*spu1* is unknown because it encodes no transposases.pSPUR2 is a 73,222-bp IncX1 plasmid with average G+C content of 47.6%. Compared to the representative IncX1 plasmid pOAL52 (51), pSPUR2 possesses a similar backbone region but shows significant differences in the accessory region. The genes carried in the backbone region are responsible for plasmid maintenance, including *hns*, *hha*, and *topB* for gene expression regulation, *par* for partitioning, *pilx* and *taxABC* for conjugal transfer, *stbED* for stability, and *pir* for replication. The accessory region exhibits a mosaic structure filled with transposable elements (TEs). A putative composite transposon flanked by IS*4* sequences contains the first of the two arsenic resistance clusters *arsB-arsC*-*arsH-arsR* and sulfonamide resistance gene *sul2* (inducing no phenotypic resistance). Another arsenic resistance cluster containing six genes, *arsP-arsR-arsH-sigL-arsD-arsA*, is located downstream. This cluster is close to the *ars2* operon in IncI1 plasmid R64 (96.9% nucleotide identity) (52). pSPUR2 also contains a Tn*2* transposon which carries the *bla*_TEM-1b_ gene, conferring resistance to penicillins and the first-generation cephalosporins (53). Within the transposase gene of Tn*2*, there is a Tn*3* family transposon (sharing 99.9% identity with Tn*As1*). In some strains, an IS*spu1*-like insertion element is present near Tn*As1*, containing at the other end a duplicated identical 1,752-bp sequence from the transposase gene of Tn*As1*.

T346Hunter (54) indicated that pSPUR1 and pSPUR2 contain a Trw-type T4SS and a VirB/D4-type T4SS, respectively, which are likely to make the plasmids self-transmissible (55). The conjugal transfer experiments were conducted by coincubating the donor strains with recipient strain Escherichia coli J53. The conjugants can be recovered for both plasmids, especially for IncN plasmid pSPUR1, which has high conjugation efficiency (Fig. S5). The results demonstrated that both plasmids are self-transmissible.

10.1128/mSphere.00627-18.5 FIG S5 Conjugation experiments of IncN plasmid pSPUR1 (left) and IncX1 plasmid pSPUR2 (right). The azide-resistant mutant of Escherichia coli was used as the recipient, and the conjugants were grown on MacConkey agar. Download FIG S5, TIF file, 0.7 MB. Copyright © 2019 Hu et al. 2019 Hu et al. This content is distributed under the terms of the Creative Commons Attribution 4.0 International license.

## DISCUSSION

A whole-genome-based typing method has been applied to the epidemiological investigation of several S. enterica serovars and has proven to be an “ultimate” approach for its maximum discriminatory power and reliable epidemiological concordance (16, 17, 20, 56–58). In the present study, we used 6,795 core genome SNPs for typing 97 *S*. Pullorum strains at the isolate-specific level and defined four phylogenetic lineages. The results not only represent the most elaborate evolutionary diagram to date but also make it possible to unify the previous typing methods. Crichton and Old previously used biochemical reactions and fimbrial-gene probes to group *S*. Pullorum, revealing that group III, an anaerogenic *S*. Pullorum group, is unable to ferment rhamnose and xylose and that it hybridizes weakly with the type-1 fimbrial subunit gene of *S*. Typhimurium (11). This finding makes sense in light of the genotype of lineage III, which has inactivated l-rhamnose isomerase gene *rhaA* and has deleted type-1 fimbrial subunit genes *fimA* and *fimI*, suggesting that lineage III is in the same phylogenetic group as group III. Also, Li et al. used multilocus enzyme electrophoresis (MLEE) to classify S. Pullorum into seven electrophoretic types (ETs), among which Pu1, Pu3, and Pu4 harbor a missense variant in *fliC*, leading to the conversion of codon 431 from alanine to threonine (6). This is a lineage-exclusive mutation of lineage III, indicating that all three of those ETs belong to lineage III. There are other genomic characteristics which could be associated with certain phenotypic traits, although we did not confirm them experimentally. For example, the glycosyltransferase operon *gtrABC* introduced by φSPU1 may potentially be involved in the antigenic variation of O12 (59). Similarly, the differences in phage types (60) could probably be attributed to the acquisition of various prophages.

The MRCA of *S*. Pullorum has been estimated to have originated around 914 CE, nearly a century before the first recognition of this pathogen in 1899 by Rettger (61). Our spatiotemporal Bayesian framework estimated that the two intercontinental transmissions of *S*. Pullorum (lineage III and IV) in modern history could be traced back to the mid-19th century and might involve a forgotten historical event dubbed Hen Fever, an economic bubble created by chicken fascination which occurred from approximately the 1840s to 1850s. The story began in 1842 when Queen Victoria received seven exotic Cochin fowls shipped from China and was deeply attracted to them. Soon, the hobby of keeping and breeding Asian chickens spread from royalty to the general population and from the United Kingdom to Europe and, finally, to the United States, culminating in the pomp of the Boston Poultry Show of 1849, in which more than 1,400 ornamental birds imported from Asia were exhibited (21). Although it seems ridiculous and inexplicable, the Hen Fever phenomenon has actually promoted chicken breeding and exchange of breeds. One example is the large chicken breed “Brahma,” which was developed in the United States by cross breeding with Chinese “Shanghai” birds. In 1852, nine gray Brahmas were exported to England and were developed into chickens of the dark Brahma variety by English breeders, which were later re-exported to the United States (62). We inferred that the time of lineage III introduction from China to Europe was 1837 (95%CI, 1782 to 1889) and that the time of lineage IV introduction to Europe from the Americas was 1842 (95%CI, 1778 to 1902), very consistent with the time when Hen Fever occurred. These data prove the accuracy and reliability of the Bayesian model used in our study, which were also supported by the coincidence of the effective population decline of lineage I within two periods: the century of humiliation in China starting from the 1850s, and the introduction of antibiotics in poultry feeding after the 1970s.

Inactivation of genes involved in metabolism and host interaction is commonly observed in host-restricted salmonellae and is considered to represent the genetic mechanism of abandonment of an intestinal colonization lifestyle and enhancement of systemic invasiveness by avoidance of nonspecific immune responses. Our study found that the genome degradation of *S*. Pullorum did not stop completely after the development of host adaptation. After diverging from the MRCA, four lineages independently accumulated pseudogenes and gene deletions, some of which have dramatically altered the bacterial phenotypes and can be used as markers for typing. It has previously been assumed that *S*. Pullorum is heading for extinction due to the irreversible accumulation of deleterious mutations, representing the hypothesis of Muller’s ratchet (34). However, our study results do not support this hypothesis. First, among most metabolic pathways and substrate transport systems with multiple alternatives, at least one remains intact in *S*. Pullorum. For instance, among the three osmoprotectant transport systems, ProU and OsmU have been inactivated in the MRCA of S. Pullorum, while ProP is highly conserved in all the strains. Similar situations are also apparent in the T3SS effectors, where *S*. Pullorum has lost the *sifB* gene required for formation of *Salmonella*-induced filaments (Sifs) but maintains its paralog *sifA*. Second, there are rare but vital spontaneous pseudogene reversions in *S*. Pullorum (see Table S4 in the supplemental material). We manually inspected the reads mapped to the genes by the use of Tablet visualization software (63) in order to confirm that the pseudogene reversions are real and not caused by assembly errors. Irrefutable evidence is provided by data corresponding to sulfate permease gene *cysU*, which was prematurely terminated at codon 131 in the MRCA of *S*. Pullorum but then reverted independently in five unrelated strains by different mutations. The reversibility of pseudogenes indicates that *S*. Pullorum maintains the ability to undo lethal mutations. In fact, *S*. Pullorum has not undergone spontaneous extinction, although it is difficult to purify even using strict surveillance and elimination measures. Even though many countries are declared to be free of pullorum disease, sporadic outbreaks often occur in noncommercial chicken flocks (2). Therefore, sustained surveillance and effective interventions are still necessary for the prevention and control of pullorum disease.

Except for the nalidixic acid resistance conferred by the mutation in codon 83 (S83F) of the GyrA protein, all of the antibiotic resistance phenotypes are mediated by the plasmids in *S*. Pullorum. Among the five resistance plasmids, pSPUR1 and pSPUR2 are more potentially hazardous than the others, for they each harbor a T4SS, a versatile secretion system facilitating the horizontal transfer of the resistance plasmids to recipient bacterial cells by conjugation (64). Chicken is one of the most important food animals, as well as being a potential reservoir for many zoonotic pathogens, including Campylobacter jejuni and Escherichia coli and other serotypes of Salmonella enterica (65–67). In coinfection with *S*. Pullorum, these foodborne pathogens might receive the multidrug resistance plasmid, which would increase potential public health risks. Furthermore, T4SSs were recently established to be associated with virulence. In *Salmonella* Heidelberg, VirB/D4 T4SS enhances bacterial entry and survival in host cells by downregulating the host genes responsible for the innate immune response (68). Also, in *Bartonella*, Trw type T4SS mediates adhesion to erythrocytes and diversifies the host specificity (69). Therefore, *S*. Pullorum strains carrying T4SS may be more pathogenic and potentially more harmful to chickens. A reassuring trend is that of the discarding of the whole resistance plasmids or partial resistance gene cassettes in certain strains (see Fig. S4 in the supplemental material). Zhou et al. attributed horizontal acquisition of resistance plasmids to transient Darwinian selection (17), which is likely to be lost as a consequence of purification in an antibiotic-free environment. Like many other countries, China has attached great importance to antibiotic abuse in food animal feeding and is taking active measures against it. In August 2016, 14 departments, including the National Health Commission and the Ministry of Agriculture, jointly issued the “National Action Plan for Preventing Bacterial Drug resistance (2016 to 2020),” in order to strengthen the supervision of the use of antibiotics and phase out the use of antibiotics in food animals for growth promotion.

In conclusion, the results of this study show that the *S*. Pullorum can be classified into four lineages and that their MRCA emerged around 1100 years ago. In the mid-19th century, two intercontinental transmission events occurred, probably related to the prevalent Hen Fever at that time. Genome degradation, mainly caused by pseudogenization, has been ongoing throughout the evolution of *S*. Pullorum. In contrast, gene acquisition mediated by bacteriophage lysogeny and plasmid transfer is also common in the *S*. Pullorum genome and has given the pathogen new properties, especially the multidrug resistance conferred by the large plasmids. The gene loss and gain in the genome of *S*. Pullorum reflect its adaptation to the changing environment. These data will be useful in further surveillance and prevention of pullorum disease.

## MATERIALS AND METHODS

### Bacterial isolates and sequencing.

A total of 97 *S*. Pullorum strains were selected from all the isolates (*n* = 655) obtained by our laboratory between 1962 and 2014 (9). The temporal and geographical origins, as well as biochemical and drug resistance phenotypes, were taken into account to encompass broad epidemiological and phylogenetic diversity. Most strains originated in China (*n* = 81), with 75 isolates from the eastern and central regions (Jiangsu, Shandong, Anhui, Henan, and Beijing) and 5 isolates from the western region (Shaanxi, Sichuan, and Xinjiang). The foreign strains (*n* = 16), including the isolates from Brazil (*n* = 5), the United Kingdom (*n* = 4), and Denmark (*n* = 2), were kindly donated by P. Barrow. The detailed metadata are listed in Table S1 in the supplemental material. Previous serological assay and ornithine decarboxylase testing were performed on all strains to confirm that they were *S*. Pullorum. Genomic DNA was prepared from overnight cultures by using DNeasy blood & tissue kits (Qiagen, Germany) according to the manufacturer’s instructions. The DNA was then fragmented with an insertion size of ∼500 bp to prepare the library and was sequenced by the use of a HiSeq 2500 system (Illumina, USA). More than 3 million paired-end (PE) reads with a length of 100 bp or 150 bp were produced for each genome. Reads with <90% Q30 bases were considered of low quality and discarded. In order to save computing resources for subsequent analyses, the filtered data were downsampled to 1.6 million reads for 150 PE or 2.4 million reads for 100 PE, with a theoretical coverage of ∼100×.

10.1128/mSphere.00627-18.6 TABLE S1 Metadata of strains used in this study. Download Table S1, XLSX file, 0.02 MB. Copyright © 2019 Hu et al. 2019 Hu et al. This content is distributed under the terms of the Creative Commons Attribution 4.0 International license.

10.1128/mSphere.00627-18.7 TABLE S2 Antibiogram achieved from both genotype and phenotype. Download Table S2, XLSX file, 0.02 MB. Copyright © 2019 Hu et al. 2019 Hu et al. This content is distributed under the terms of the Creative Commons Attribution 4.0 International license.

10.1128/mSphere.00627-18.8 TABLE S3 Estimation of emergence dates and clock rates. Download Table S3, XLSX file, 0.01 MB. Copyright © 2019 Hu et al. 2019 Hu et al. This content is distributed under the terms of the Creative Commons Attribution 4.0 International license.

10.1128/mSphere.00627-18.9 TABLE S4 Pseudogenes and gene deletions. Download Table S4, XLSX file, 0.1 MB. Copyright © 2019 Hu et al. 2019 Hu et al. This content is distributed under the terms of the Creative Commons Attribution 4.0 International license.

### Core-genome single nucleotide polymorphism (SNP) calling.

The genome of *S*. Pullorum RKS5078 (GenBank accession no. CP003047) (42) was used as the reference. We defined the core genome as a reference genome that excludes repeat, deletion, and recombination regions. Repeats can cause ambiguities and affect the accuracy of alignment and assembly (70). Four criteria and methods were used to identify the following repeat regions: (i) duplicated regions of >50 bp identified by using NUCmer (71) to align the reference genome with itself; (ii) the regions of copy number variations (CNVs) inferred by using both runHMM and runBootstrap methods in the R package of CNOGpro (72); (iii) variable number tandem repeats (VNTRs) and low-complexity regions recognized by using RepeatModeler (73); and (iv) RNA and CRISPR elements recognized by using Prokka (74). Deletions provided null information. A custom script (*dsvfinder.py*) was used to recognize deletion structural variations. Briefly, after *de novo* assembling from reads by using SPAdes (75), the draft genome was aligned with the reference genome. The matched regions in reference genome were subtracted by using BEDTools (76) to fetch the deletions. For short deletions, we used *breseq* (77), a read-mapping-based tool which was sensitive to indels shorter than read length and was also accurate in identifying the boundaries of deletions in large-scale structural variations (SVs). Theoretically, the presence of recombination can distort the branch length of a phylogenic tree (78). We used Gubbins (15) to detect recombination regions from the alignment of genomic consensus sequences with 10 iterations.

Paired reads of each strain were mapped to the reference genome by using Bowtie2 (79), and Freebayes (80) was then used to call the SNPs from the BAM files. The SNPs with a coverage level of <10 or mapping quality value of <30 were excluded. A homemade Python script was used to apply the mutations to the reference genome, in order to generate a consensus sequence for each strain. After masking of the noncore regions with Ns, the core genome SNPs were extracted by using *SNP-sites* (81).

### Phylogenetic analysis.

The population structure was predicted from the SNP data set by using fastSTRUCTURE (18) and validated in combination with the cladogram. The optimal substitution model was inferred from the alignment of SNPs by using jModelTest 2 (82) and then used for maximum likelihood (ML) and Bayesian phylogenetic analyses. The ML phylogenetic tree was constructed by using RAxML (83) with GTR modeling. A total of 1,000 rapid bootstrap inferences and thereafter a thorough ML search were carried out to find the best ML tree. *S*. Gallinarum was used as an outgroup to root the tree.

BEAST 1.8.4 (84) was used to perform temporal Bayesian phylogenetic analysis on the time-stamped SNP data set, including inferring the phylogenetic tree, estimating the date of the most recent common ancestor (MRCA), calculating the evolutionary rate, and reconstructing the evolutionary dynamics. Strains with an unknown isolation time were assigned to a specific taxon set and sampled with individual priors in order to estimate the date. All the strains of lineage I to III were assigned to a single monophyletic taxon set in order to root the tree correctly. In the initial phase, six combinations of two clock models (strict clock and relaxed lognormal clock) and three population models (constant size, Bayesian SkyGrid, and Bayesian Skyline) were tested beforehand. For each combination, Markov chain Monte Carlo (MCMC) analysis of 100 million states was run, with sampling every 10,000 iterations, after which both path sampling (PS) and stepping-stone sampling (SS) methods were performed to estimate the marginal likelihood and compare the model results. The highest Bayes factor was obtained for the combination of relaxed lognormal clock and constant population size models, on which an ultimate run was executed, with the length of chain extending to 600 million states. We repeated this process on lineage I, which was the preponderant Chinese lineage in our data set, and found that relaxed lognormal clock and Bayesian SkyGrid are the optimal models for this subset. The final maximum clade credibility (MCC) tree was generated by TreeAnnotator and visualized by iTOL (85).

### Identification of pseudogenes and gene deletions.

A reference CDS set was established by parsing the NCBI GenBank records of *S*. Enteritidis P125019 (accession no. NC_011294) and *S*. Gallinarum 287/91 (accession no. NC_011274). All the CDS and pseudo-CDS entries were extracted, and the redundant ones were excluded by retrieving the protein IDs and curating manually. We also included the CDSs from the virulence plasmid of *S*. Pullorum ATCC 9120 (accession no. CP012348) for it was conserved in all the *S*. Pullorum strains that we examined.

We defined a pseudogene as a homologous locus with >10% in-frame codons affected by the mutations compared to its reference. The mutations included premature termination codons, frameshifts, in-frame insertions and deletions, and disrupted initiation codons. We wrote a script (*orthoanno.py*) to identify the pseudogenes. In brief, the script aligned the protein sequences translated from the reference CDSs to each draft genome by tblastn (86) in order to locate the orthologous gene in the subject genome with a cutoff identity of ≥95% and expected value of ≥1E−10. The located genes with the exact boundaries were then translated and checked for premature termination codons (PTC) and frameshift mutations. Furthermore, a read-mapping-based method was used to inspect the accurate mutation site in pseudogenes. In short, variants compared to the reference genome in each strain were called by *breseq* and dumped to the VCF records. SnpEff v4.3 (87) was then used to annotate the effects of the variants on genes at both the DNA and protein levels.

### Identification of mobile genetic elements and antimicrobial resistance genes (ARGs).

We used *dsvfinder.py* to extract the regions of difference (RODs), which are potentially related to mobile genetic elements (22). Then, the RODs were searched in the PHAST (88) and PlasmidFinder (49) databases for detection and typing prophages and plasmids. For the prophages, the attachment sites were manually inspected by searching for the flanked repetitive sequences. To reorder and circularize the fragments of plasmids, the overlapped sequences at both ends were considered and Sanger sequencing was performed when necessary. The hypothetical transposable elements annotated by Prokka were submitted to ISfinder (89) for typing and determining the boundaries.

Two scripts, *argfinder.py* and *mutfinder.py*, were written to identified acquired antimicrobial resistance genes (ARGs) and point mutations, respectively. The *argfinder.py* script used a two-step Blast strategy to locate the ARGs and to find the best hit matches in the Resfinder database (90) (which contains redundancy). The records with identity levels of <90% or coverage levels of <60% were discarded. The *mutfinder.py* script used blastn to aligned four genes (*gyrA*, *gyrB*, *parC*, and *parE*) involved in fluoroquinolone resistance to the draft genomes, and then the matched nucleotide sequences were translated and the codons were compared pairwise to find the point mutations. The impacts of gene acquisitions and mutations were interpreted according to the comprehensive antibiotic resistance database (91) and reference articles.

### Plasmid conjugal transfer assay.

The liquid cultures of donor strain 1204 (pSPUR1) or 20-56 (pSPUR2) and (azide-resistant) recipient strain Escherichia coli J53 were mixed and coincubated overnight. The mixture was placed on MacConkey agar with the addition of 200 μg/ml sodium azide and 200 μg/ml tetracycline for selecting conjugants.

### Biochemical assays and antimicrobial susceptibility testing.

The biochemical assays were executed using API 20E microbial identification kits (bioMérieux, France), and the results were interpreted according to the manufacturer’s instructions. The MIC was determined by using a miniaturized broth dilution test described previously (92) and interpreted according to the breakpoint tables published by the Clinical and Laboratory Standards Institute.

### Data availability.

The clean reads of sequencing strains used in this study have been deposited in the European Nucleotide Archive (ENA) under project accession no. PRJEB27755. The accession numbers for each strain are listed in Table S1. The annotated assemblies for plasmid pSPUR1 and plasmid pSPUR2 are available at ENA under accession numbers LR536428 and LR536427, respectively. The custom scripts are available at GitHub (https://github.com/yachenhu/Salmonella_pullorum_evolution) for public download.

10.1128/mSphere.00627-18.10 TABLE S5 Degraded metabolic pathways and transport systems affected by gene inactivation. Download Table S5, XLSX file, 0.01 MB. Copyright © 2019 Hu et al. 2019 Hu et al. This content is distributed under the terms of the Creative Commons Attribution 4.0 International license.
