# Supplementary figures and images for: Loss and Gain in the Evolution of the Salmonella enterica Serovar Gallinarum Biovar Pullorum Genome
Source: mSphere. 2019 Apr 3;4(2):e00627-18. doi: 10.1128/mSphere.00627-18 (PMC6449608; doi:10.1128/mSphere.00627-18)

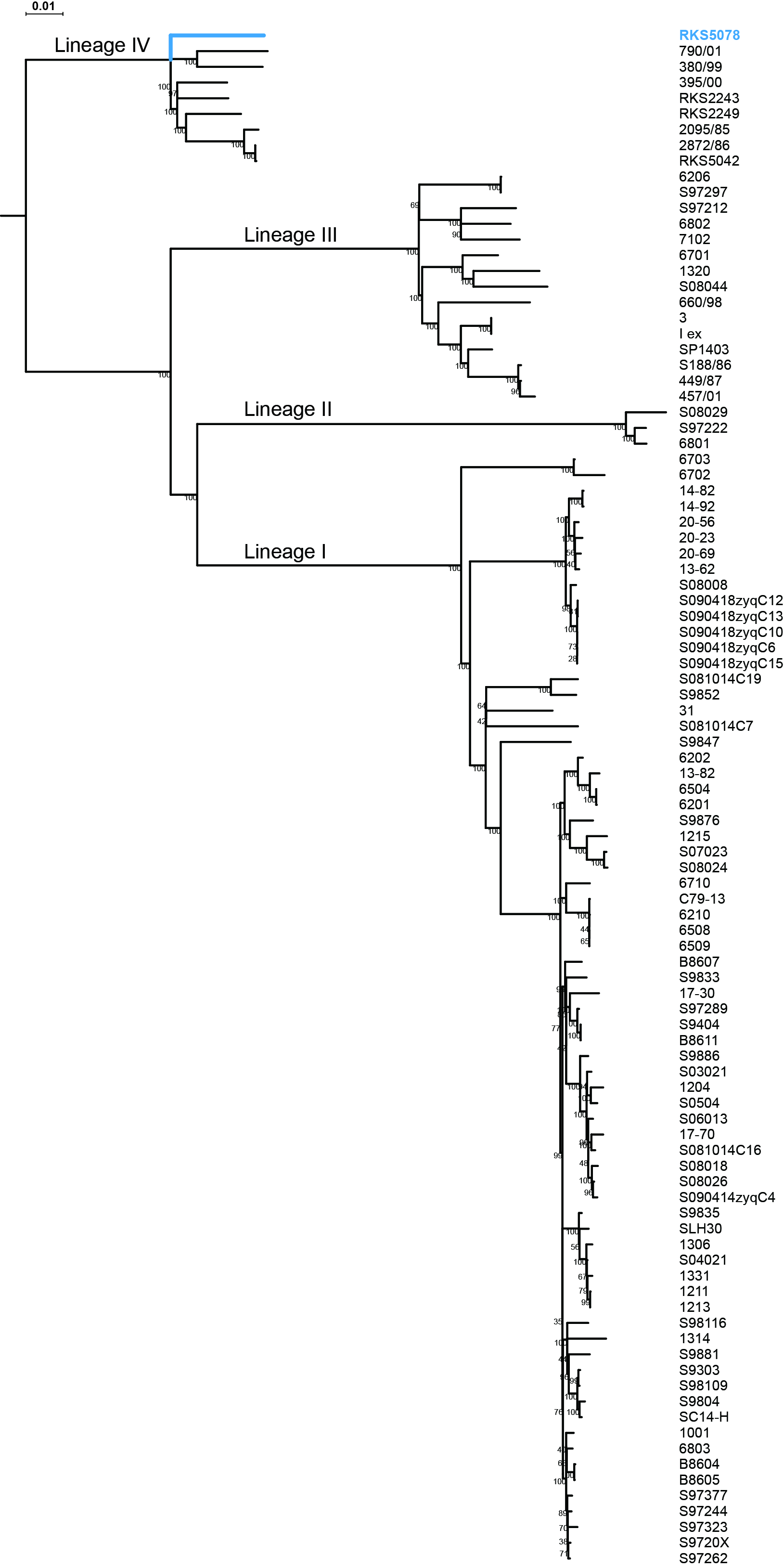

Supplement: FIG S1 [file mSphere.00627-18-sf001.tif]

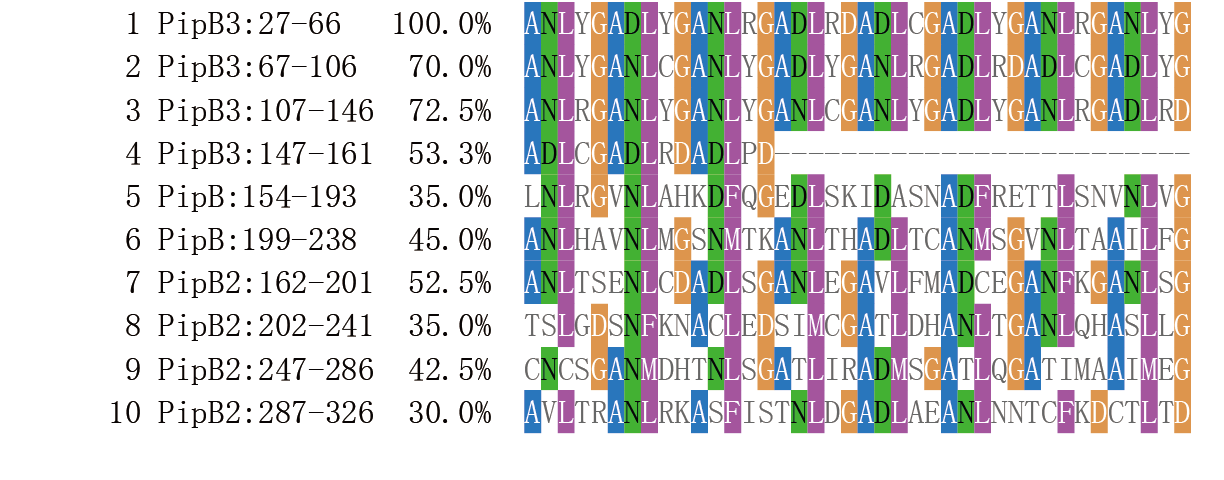

Supplement: FIG S3 [file mSphere.00627-18-sf003.tif]

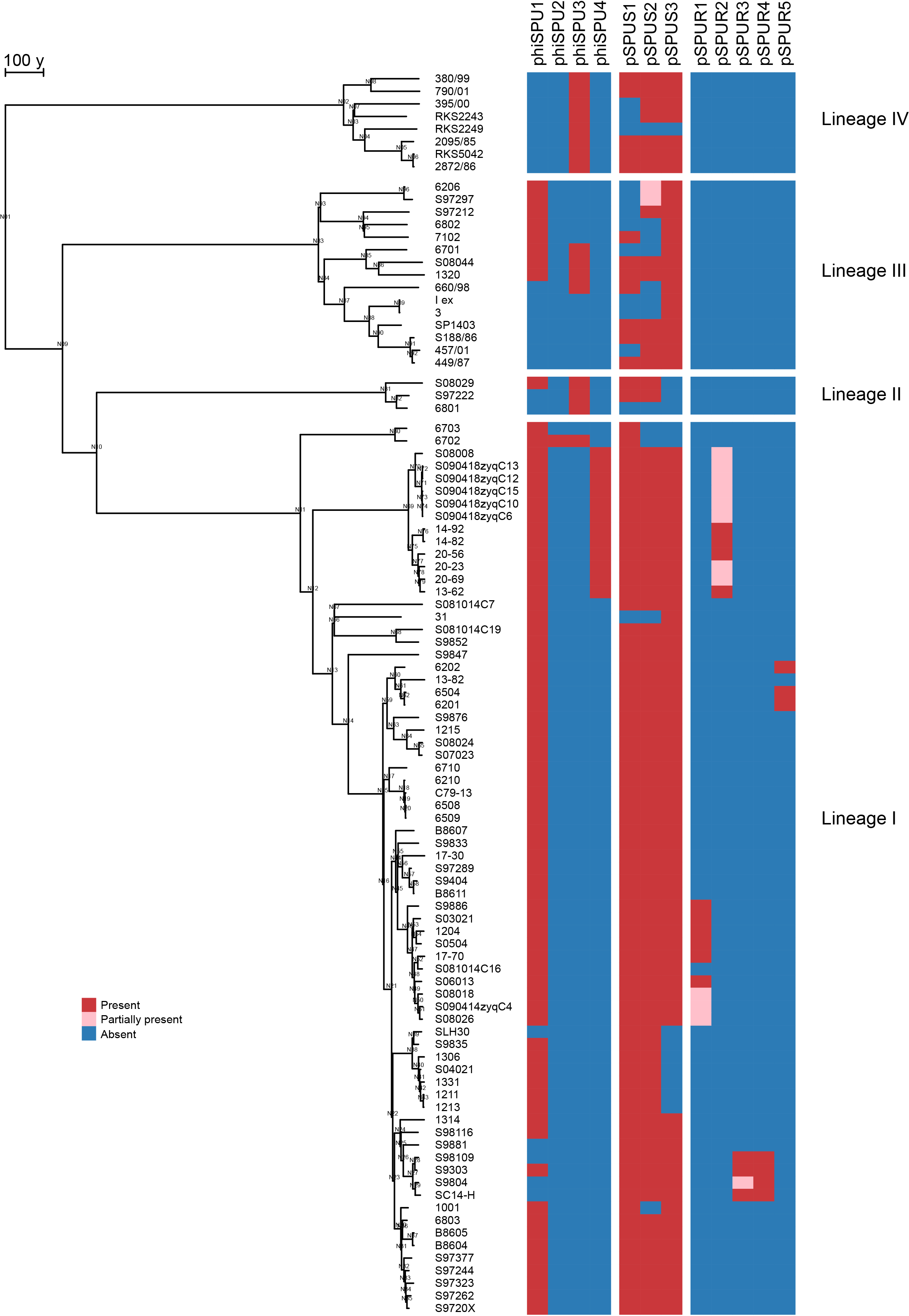

Supplement: FIG S4 [file mSphere.00627-18-sf004.tif]

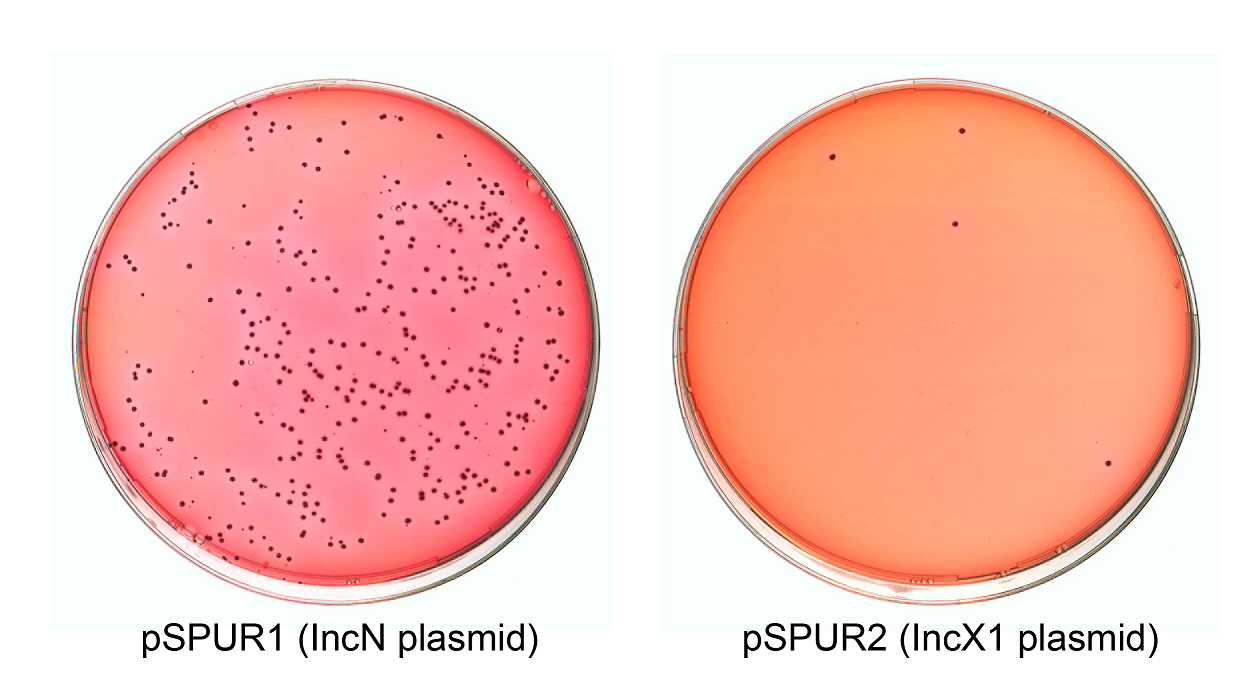

Supplement: FIG S5 [file mSphere.00627-18-sf005.tif]
